# eIF4E-Dependent Translation Potentially Regulates Apoptosis and BDNF/TrkB Signaling in the Medial Prefrontal Cortex During Morphine-Induced CPP

**DOI:** 10.3390/ijms27115097

**Published:** 2026-06-04

**Authors:** Feifei Gao, Xixi Yang, Yuyanran Zhang, Dongyu Yu, Jie Chen, Beilin Hou, Zhuojin Yang, Lanjiang Li, Danmei Wang, Shaqin Xie, Danni Gao, Xin Liu, Hongrui Qian, Yuxiang Zhang, Chunxia Yan

**Affiliations:** 1 Key Laboratory of Forensic Medicine of National Health Commission, College of Forensic Medicine, Xi’an Jiaotong University Health Science Center, Xi’an 710061, China; gff1223@stu.xjtu.edu.cn (F.G.); yangxixi88@stu.xjtu.edu.cn (X.Y.); 3121115147@stu.xjtu.edu.cn (D.Y.); chenjie777@stu.xjtu.edu.cn (J.C.); houbeilin@stu.xjtu.edu.cn (B.H.); cloris_sunshine@stu.xjtu.edu.cn (Z.Y.); dyjc17@stu.xjtu.edu.cn (D.W.); xsq-51129@stu.xjtu.edu.cn (S.X.); 15664955802@163.com (D.G.);; 2 Bio-Evidence Science Academy, Science and Technology Innovation Harbour of Western China, Xi’an Jiaotong University, Xi-Xian New Area Fengxi New City, Xi’an 712000, China; 3 Key Laboratory of Shaanxi Province for Craniofacial Precision Medicine Research, College of Stomatology, Xi’an Jiaotong University, Xi’an 710004, China; zhangnemo@stu.xjtu.edu.cn; 4 Key Laboratory of Drug Addiction Medicine of National Health Commission, School of Forensic Medicine, Kunming Medical University, Kunming 650504, China; lilanjiang@kmmu.edu.cn; 5 National Narcotics Laboratory Shaanxi Branch, Xi-Xian New Area Fengxi New City, Xi’an 712000, China

**Keywords:** morphine, reward memory, eukaryotic translation initiation factor 4E, medial prefrontal cortex, apoptosis, brain-derived neurotrophic factor

## Abstract

Morphine addiction is driven in part by persistent reward-associated memory, yet the molecular mechanisms linking translational control to cellular stress responses remain unclear. In the present study, using a mouse morphine-induced conditioned place preference and N2a cell model, we investigated apoptosis-related alterations in the medial prefrontal cortex and the involvement of eIF4E-dependent translational regulation and BDNF/TrkB signaling. Morphine-induced conditioned place preference was associated with an increase in TUNEL-positive cells in the medial prefrontal cortex, accompanied by upregulation of Bax and downregulation of Bcl-2. In N2a cells, morphine induced apoptosis in a dose-dependent manner. Morphine also increased neuronal eIF4E expression in both mPFC tissue and N2a cells, accompanied by upregulation of BDNF and TrkB. Inhibition of the eIF4E/eIF4G interaction with 4EGI-1 significantly affected morphine-induced CPP formation and altered apoptosis-related markers and BDNF/TrkB expression. Notably, intra-mPFC administration of 4EGI-1 suppressed morphine-induced CPP without affecting anxiety-like behavior, locomotor activity, or general learning and memory performance. These findings suggest that eIF4E-dependent translational regulation is functionally associated with morphine-induced reward memory and apoptosis, potentially in association with changes in BDNF/TrkB molecular expression. This study offers novel insight into the molecular basis of morphine addiction and highlights a potentially targetable translational regulatory pathway for therapeutic intervention.

## 1. Introduction

Drug addiction is a chronic relapsing brain disorder characterized by compulsive drug-seeking behavior, persistent drug consumption, and a strong tendency toward relapse [1]. A central pathological basis of addiction lies in the formation and long-term maintenance of aberrant associative memories linking drug-related environmental cues with rewarding experiences [2]. Growing evidence suggests that the formation, consolidation, and reactivation of drug-associated reward memory represent key neurobiological processes that drive craving and relapse [3]. Therefore, elucidating the molecular mechanisms that regulate addiction-related reward memory is essential for understanding the pathophysiology of drug addiction and identifying potential therapeutic targets.

Opioids, particularly morphine, are widely used in clinical practice because of their potent analgesic effects; however, prolonged or inappropriate use can lead to pronounced psychological dependence and addictive behaviors [4,5]. Conditioned place preference (CPP) is a classical behavioral paradigm for evaluating drug reward and drug-associated contextual memory, and it has been extensively used to investigate morphine-related reward memory [6]. Previous studies have demonstrated that morphine-induced reward memory is mediated not by a single brain region, but by the coordinated engagement of multiple reward-related areas [7,8]. Among these regions, the medial prefrontal cortex (mPFC) serves as a key hub for the integration of contextual information, reward cue processing, behavioral control, and susceptibility to relapse [9]. The mPFC participates not only in the encoding and retrieval of drug-associated cues, but also in the modulation of addictive behaviors through its regulation of downstream reward circuits [10]. Therefore, it is widely regarded as a key brain region for exploring the formation and maladaptive consolidation of morphine-associated reward memory.

Current studies on morphine-induced addiction-related plasticity have mainly focused on synaptic remodeling, neurotransmitter imbalance, and epigenetic regulation [11,12,13]. In contrast, whether reward memory formation is accompanied by cellular stress-related responses, particularly apoptosis-related responses, remains insufficiently understood. Previous studies have suggested that opioid exposure can induce apoptosis-related molecular changes, including increased Bax expression, reduced Bcl-2 expression, and caspase activation [14,15]. Although the mPFC is a critical brain region involved in reward memory and behavioral regulation, whether morphine triggers apoptosis-related responses during reward memory formation and how these responses are associated with eukaryotic translation initiation factor 4E (eIF4E)-dependent translational regulation in the mPFC remains unclear.

Translational regulation constitutes a fundamental molecular mechanism underlying neuroplasticity and memory formation [16], while cap-dependent translation initiation plays a pivotal role in synaptic remodeling [17,18]. eIF4E is a key rate-limiting factor in cap-dependent translation initiation. By binding to eIF4G to form the eIF4F complex, eIF4E regulates the translation efficiency of specific mRNAs [19]. Increasing evidence suggests that eIF4E is involved not only in learning, memory, and synaptic plasticity [20], but also in cellular stress responses, injury repair, and cell fate determination [21,22]. However, in the context of morphine-associated reward memory, it remains unclear whether eIF4E participates in molecular remodeling within the mPFC and whether it is functionally linked to apoptosis-related responses.

In addition to translational regulation, brain-derived neurotrophic factor (BDNF) and its receptor, tropomyosin receptor kinase B (TrkB), form a signaling pathway critically involved in neuronal survival, synaptic plasticity, and addiction-related neural adaptations [23,24]. Previous studies have demonstrated that BDNF/TrkB signaling contributes to opioid-induced neuroplastic changes and is closely associated with addiction-related memory and relapse-related behaviors [25]. At the same time, this pathway represents an important molecular support system for cellular stress responses and survival maintenance [26,27]. It remains unclear whether BDNF/TrkB signaling cooperates with eIF4E-dependent translational regulation in morphine-induced reward memory and cellular stress responses.

Herein, the present study employed a mouse morphine-induced CPP model and an N2a cell model to systematically investigate apoptosis-related alterations, eIF4E expression changes, and BDNF/TrkB signaling remodeling in the mPFC following morphine exposure. In addition, the eIF4E/eIF4G interaction inhibitor 4EGI-1 was used to explore the role of eIF4E-dependent translational regulation in morphine-associated reward memory formation and cellular stress responses. This study aimed to provide new experimental evidence for elucidating the molecular mechanisms underlying morphine addiction and identifying potential targets for intervention.

## 2. Results

### 2.1. Establishment of a Morphine-Induced CPP Model in Mice

**Figure 1:**
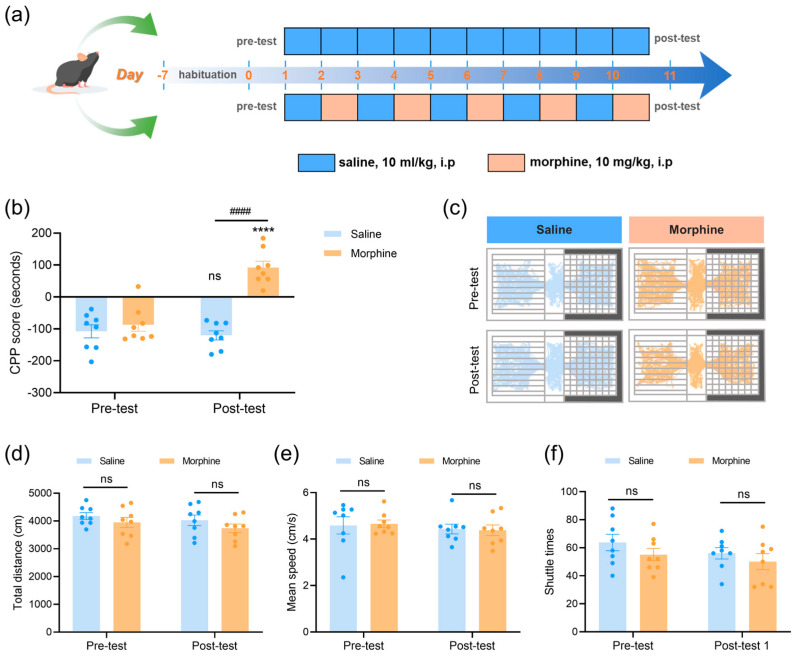
Morphine induces conditioned place preference in mice without affecting general locomotor activity. (**a**) Schematic illustration of the CPP procedure. Mice underwent habituation and pre-test, followed by conditioning sessions and a post-test. (**b**) CPP scores in saline- and morphine-treated mice during pre-test and post-test. No significant difference was observed between groups during the pre-test, whereas the morphine group showed a significantly increased CPP score in post-test. *n* = 8, **** *p* < 0.0001 compared to the pre-test, #### *p* < 0.0001 compared to the saline group. (**c**) Representative locomotor trajectories of mice from the saline and morphine groups during pre-test and post-test. (**d**–**f**) Quantification of total distance traveled, mean velocity and shuttle times in the two groups. *n* = 8, ns, not significant.

To evaluate whether morphine exposure induced stable reward-associated memory, CPP, as illustrated in Figure 1a, was employed. During the pre-test phase, no significant difference in CPP scores was detected between the saline and morphine-treated groups, indicating the absence of an intrinsic chamber bias before conditioning. After repeated conditioning sessions, mice in the morphine-treated group exhibited a marked increase in CPP score in the post-test compared with the saline group (*F* = 64.737, *p* < 0.0001), indicating a strong preference for the morphine-paired chamber (Figure 1b). These findings indicate that morphine successfully induced a stable context-dependent reward memory in mice. Representative locomotor trajectories further supported this behavioral phenotype (Figure 1c).

Locomotion-related parameters were further analyzed to determine whether the altered chamber preference was attributable to nonspecific changes in locomotor activity. No significant difference in total distance traveled, mean velocity, or shuttle times were detected between saline- and morphine-treated group (Figure 1d–f). These results suggest that the increased CPP score in morphine-treated mice was not attributable to changes in general locomotor capacity or exploratory behavior, but rather reflected the successful formation of morphine-associated reward memory.

Collectively, these data demonstrate that a reliable morphine-induced CPP mouse model was successfully established, providing a robust behavioral foundation for subsequent mechanistic investigations into reward memory.

### 2.2. Morphine Promotes Apoptotic-Related Responses and Enhances eIF4E and BDNF/TrkB Expression in the mPFC

Accumulating evidence indicates that persistent drug exposure not only remodels synaptic function within reward-related brain regions, but may also trigger maladaptive cellular responses, including apoptosis, which in turn contribute to the formation and maintenance of addiction-associated neuroplasticity [28]. Given the critical role of the mPFC in reward memory and behavioral control [29], apoptotic alterations in this region were further examined following morphine exposure.

**Figure 2:**
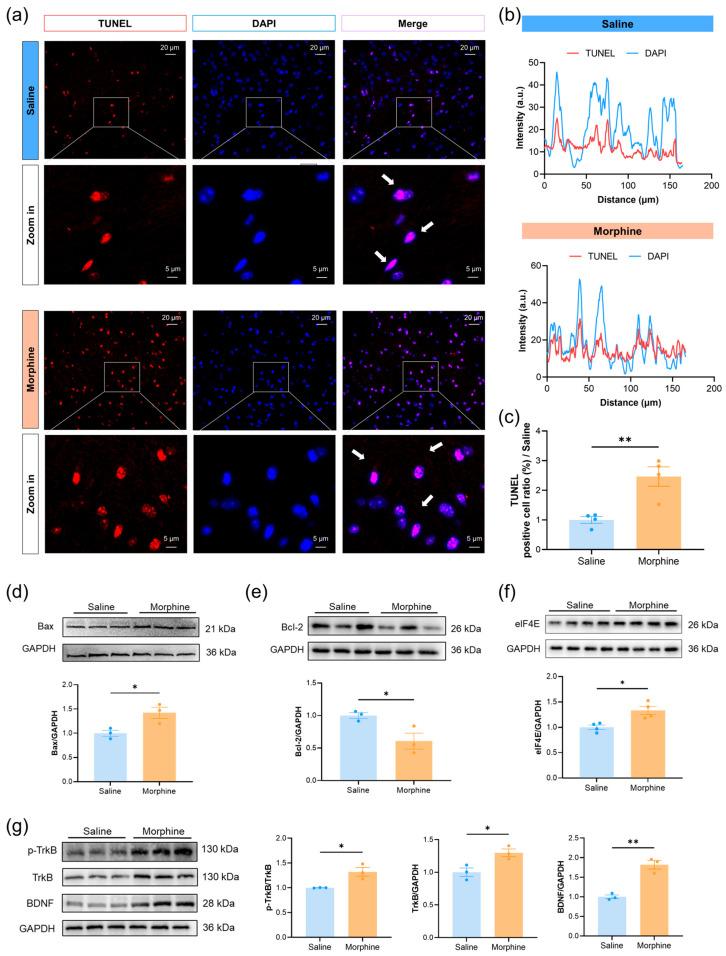
Morphine exposure increases apoptotic-related responses and upregulates eIF4E and BDNF/TrkB expression in the mPFC. (**a**) Representative TUNEL staining images of the mPFC from saline- and morphine-treated mice. TUNEL-positive cells are shown in red, and nuclei are counterstained with DAPI (blue). Merged images and corresponding enlarged views are shown. White arrows indicate TUNEL-positive nuclei. Upper: scale bar = 20 μm, zoomed: scale bar = 5 μm. (**b**) Colocalization analysis of TUNEL-positive signals with DAPI staining in the mPFC of saline- and morphine-treated mice. (**c**) Morphine treatment significantly increased the percentage of TUNEL-positive cells in the mPFC. *n* = 4, ** *p* < 0.01 compared to the saline group. (**d**) Western blot analysis and quantification showing that morphine significantly increased neuronal Bax protein expression in the mPFC. *n* = 3, * *p* < 0.05 compared to the saline group. (**e**) Western blot analysis and quantification showing that morphine significantly decreased neuronal Bcl-2 protein expression in the mPFC. *n* = 3, * *p* < 0.05 compared to the saline group. (**f**) Western blot analysis and quantification showing that morphine significantly increased neuronal eIF4E protein expression in the mPFC. *n* = 4, * *p* < 0.05 compared to the saline group. (**g**) Western blot analysis and quantification showing that morphine significantly increased neuronal BDNF, total TrkB, and p-TrkB expression in the mPFC. *n* = 3, * *p* < 0.05, ** *p* < 0.01 compared to the saline group.

TUNEL staining revealed that only a limited number of TUNEL-positive cells were present in the mPFC of saline-treated mice, whereas morphine-treated mice exhibited a marked increase in TUNEL-positive signals (Figure 2a). Enlarged images further showed a greater number of TUNEL-positive nuclei in the morphine group. Consistent with this observation, fluorescence colocalization analysis demonstrated an increased overlap of TUNEL and DAPI signals in morphine-treated mice, supporting an elevation in apoptotic cells within the mPFC (Figure 2b). Quantitative analysis further confirmed that the percentage of TUNEL-positive cells was significantly increased after morphine treatment (Figure 2c, *t* = 4.236, *p* = 0.0055).

Neuronal proteins were isolated from the mPFC using neuron-specific protein extraction reagent to examine apoptosis-related molecular alterations. Morphine treatment significantly increased the expression of the pro-apoptotic protein Bax (Figure 2d, *t* = 3.196, *p* = 0.0330), while reducing the level of the anti-apoptotic protein Bcl-2 (Figure 2e, *t* = 3.033, *p* = 0.0387). In parallel, eIF4E protein expression was also markedly upregulated in the morphine group (Figure 2f, *t* = 3.639, *p* = 0.0109). Given the important role of BDNF/TrkB signaling in neuronal survival and reward-related molecular adaptation, we further examined TrkB, phosphorylated TrkB (p-TrkB) and BDNF protein expression in the mPFC. Western blot analysis showed that morphine treatment significantly increased total TrkB (*t* = 3.371, *p* = 0.0280) and BDNF (*t* = 6.903, *p* = 0.0023) expression compared with the saline group. Importantly, morphine also increased the level of p-TrkB (*t* = 3.653, *p* = 0.0217), indicating that morphine-induced CPP formation was accompanied by functional activation of TrkB-related signaling in the mPFC. (Figure 2g).

These results demonstrate that morphine exposure induces apoptotic-related responses in the mPFC, as evidenced by increased TUNEL-positive cells, elevated neuronal Bax expression, and reduced neuronal Bcl-2 expression. Moreover, the concomitant upregulation of eIF4E, TrkB, p-TrkB and BDNF suggests that morphine-induced reward memory formation is associated with coordinated molecular alterations involving apoptosis-related responses, translational regulation, and BDNF/TrkB signaling in mPFC neurons.

### 2.3. Morphine Induces Apoptotic-Related Responses in N2a Cells

**Figure 3:**
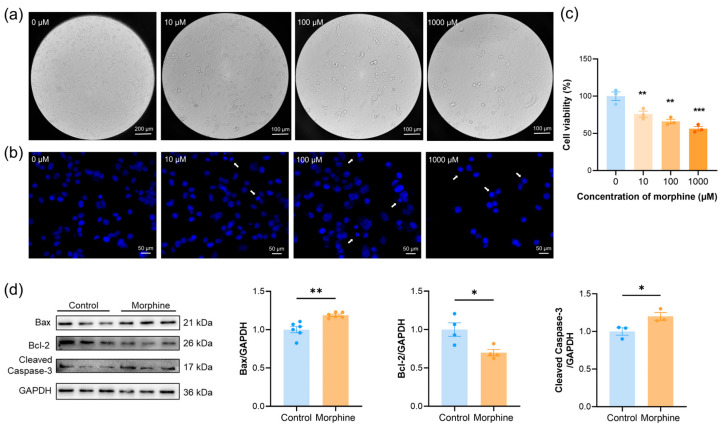
Morphine induces apoptotic injury in N2a cells. (**a**) Representative bright-field images showing concentration-dependent morphological injury in N2a cells after 48 h morphine treatment. Scale bar, 200 μm in the 0 μM group and 100 μm in the other groups. (**b**) Representative DAPI staining images showing increased nuclear condensation and fragmentation after morphine treatment for 48 h. White arrows indicate nuclei with apoptotic morphology. Scale bar = 50 μm. (**c**) Cell viability analysis showing that morphine significantly reduced N2a cell viability in a dose-dependent manner after 48 h exposure, with significant decreases observed at 10 μM (*t* = 4.209, *p* = 0.0089), 100 μM (*t* = 5.927, *p* = 0.0011), and 1000 μM (*t* = 7.638, *p* = 0.0002) compared with the 0 μM control group. ** *p* < 0.01, *** *p* < 0.001, *n* = 3. (**d**) Representative Western blots and quantitative analysis of apoptosis-related markers in N2a cells following 1000 μM morphine treatment. Morphine increased Bax and cleaved caspase-3 expression while decreasing Bcl-2 expression. *n* = 3–6, * *p* < 0.05, ** *p* < 0.01 compared to the 0 μM control group.

Morphine was applied to N2a cells at increasing concentrations (0, 10, 100, and 1000 μM) for 48 h to further validate its pro-apoptotic effect in vitro. Bright-field imaging showed concentration-dependent morphological injury, including reduced cell density, cell shrinkage, and increased cellular rounding, which was most evident at 1000 μM (Figure 3a). Consistently, DAPI staining revealed increased nuclear condensation and fragmentation following morphine exposure (Figure 3b). Cell viability was significantly reduced in a dose-dependent manner after 48 h treatment (Figure 3c, *F* = 21.47, *p* = 0.0004). Western blot analysis further showed that 1000 μM morphine increased Bax expression (*t* = 4.369, *p* = 0.0014), decreased Bcl-2 expression (*t* = 3.105, *p* = 0.0210), and increased cleaved caspase-3 expression (Figure 3d, *t* = 2.809, *p* = 0.0484). These results provide additional evidence that morphine induces apoptosis-related cellular injury in N2a cells. Based on these findings, 1000 μM morphine administered for 48 h was selected for subsequent in vitro experiments.

### 2.4. Morphine Increases eIF4E, BDNF and TrkB Expression in N2a Cells

**Figure 4:**
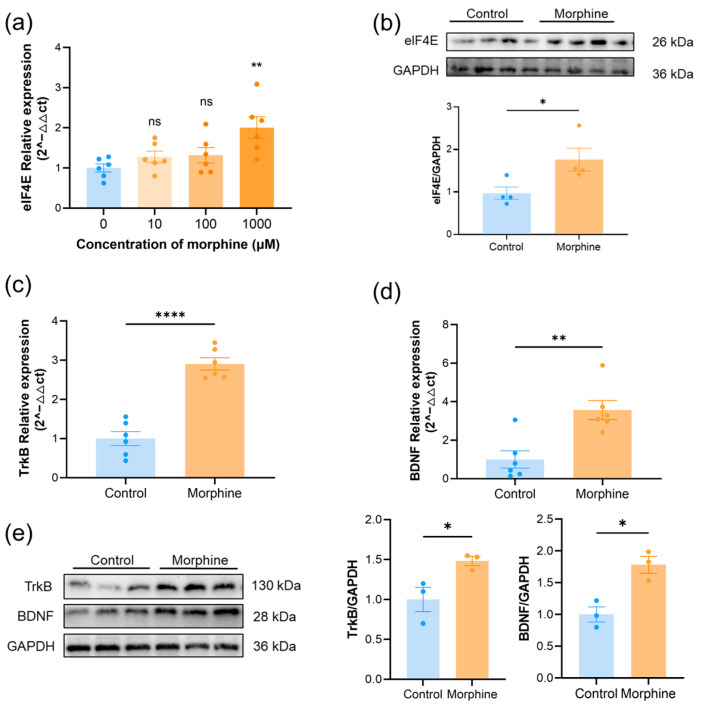
Morphine exposure increases eIF4E, BDNF, and TrkB expression in N2a cells. (**a**) qPCR analysis showing that morphine increased *eIF4E* mRNA expression in a concentration-dependent manner in N2a cells after 48 h treatment, with a significant increase observed at 1000 μM (*t* = 3.812, *p* = 0.0033) compared to the 0 μM control group. ** *p* < 0.01, ns, not significant, *n* = 6. (**b**) Western blot analysis and quantification showing that 1000 μM morphine treatment for 48 h significantly increased eIF4E protein expression in N2a cells. *n* = 4, * *p* < 0.05 compared to the control group. (**c**) qPCR analysis showing that 1000 μM morphine treatment for 48 h significantly increased *TrkB* mRNA expression in N2a cells. *n* = 6, **** *p* < 0.0001 compared to the control group. (**d**) qPCR analysis showing that 1000 μM morphine treatment for 48 h significantly increased *BDNF* mRNA expression in N2a cells. *n* = 6, ** *p* < 0.01 compared to the control group. (**e**) Western blot analysis and quantification showing that 1000 μM morphine treatment for 48 h significantly increased TrkB and BDNF protein expression in N2a cells. *n* = 3, * *p* < 0.05 compared to the control group.

Morphine-induced molecular changes were further investigated in vitro by treating N2a cells with increasing concentrations of morphine for 48 h. qPCR analysis showed that *eIF4E* mRNA expression was significantly increased only at 1000 μM, whereas no significant changes were observed at 10 or 100 μM (Figure 4a, *F* = 5.254, *p* = 0.0078), suggesting that transcriptional upregulation of eIF4E may occur mainly under higher levels of morphine-induced cellular stress. Based on this result, 1000 μM morphine was selected for subsequent experiments. Using this condition, Western blot analysis confirmed that morphine significantly increased eIF4E protein expression in N2a cells (Figure 4b, *t* = 2.581, *p* = 0.0417). Meanwhile, qPCR analysis showed that morphine significantly upregulated the mRNA expression of both *TrkB* (*t* = 8.001, *p* < 0.0001) and *BDNF* (*t* = 3.840, *p* = 0.0033) (Figure 4c,d). Consistently, protein testing further demonstrated that morphine significantly increased the protein levels of TrkB (*t* = 2.951, *p* = 0.0419) and BDNF (*t* = 4.348, *p* = 0.0122) (Figure 4e). These findings demonstrate that 1000 μM morphine enhances eIF4E expression and increases BDNF and TrkB expression in N2a cells.

### 2.5. 4EGI-1 Aggravates Morphine-Induced Apoptotic-Related Responses in N2a Cells

**Figure 5:**
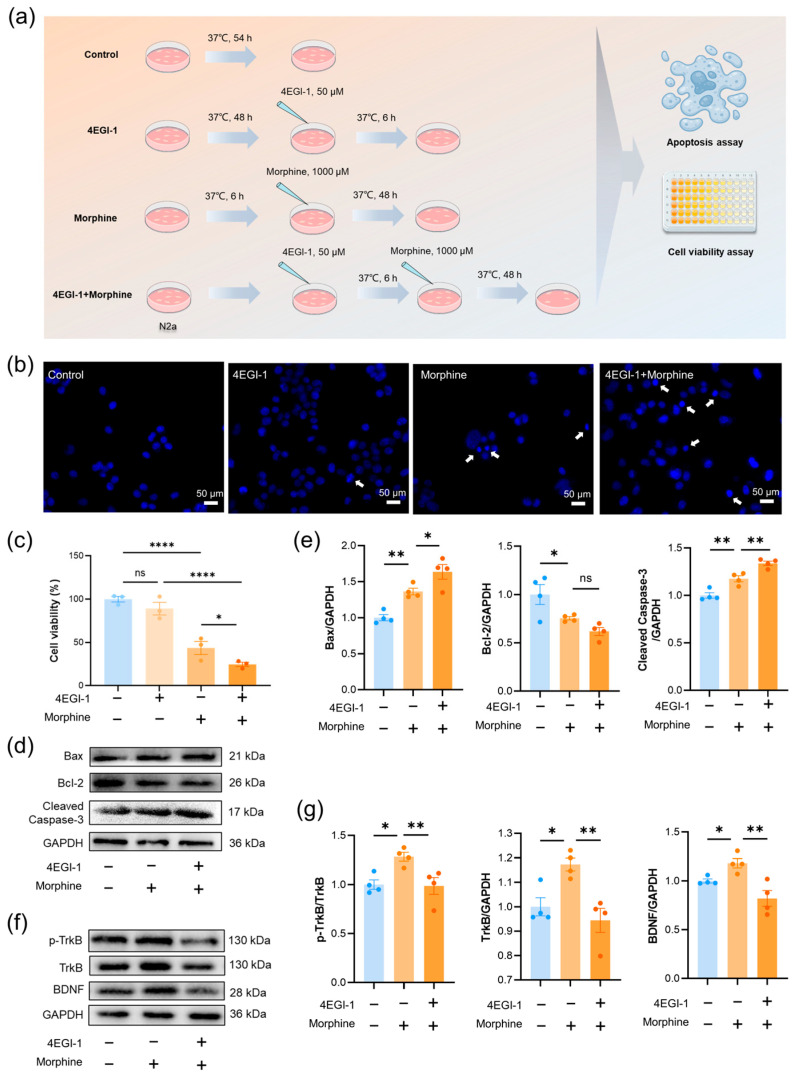
4EGI-1 aggravates morphine-induced apoptotic injury and attenuates BDNF and TrkB expression in N2a cells. (**a**) Schematic illustration of the experimental design. N2a cells were treated with 4EGI-1 (50 μM), morphine (1000 μM), or their combination, followed by apoptosis, cell viability assays and Western blotting. (**b**) Representative DAPI staining images showing that morphine induced apoptotic nuclear changes in N2a cells, which were further aggravated by cotreatment with 4EGI-1. Blue fluorescence indicates DAPI-stained nuclei, and white arrows indicate nuclei with apoptotic morphology. Scale bar = 50 μm. (**c**) Cell viability analysis showing that 4EGI-1 alone did not significantly affect cell viability but further enhanced the morphine-induced reduction in viability. *n* = 3, * *p* < 0.05, **** *p* < 0.0001. (**d**) Representative Western blots of Bax, Bcl-2 and cleaved caspase-3 in N2a cells under the indicated treatments. (**e**) Quantitative analysis showing that morphine increased Bax and cleaved caspase-3 expression and decreased Bcl-2 expression, while 4EGI-1 pretreatment further enhanced Bax and cleaved caspase-3 expression. *n* = 4, * *p* < 0.05, ** *p* < 0.01, ns, not significant. (**f**) Representative Western blots of total TrkB, p-TrkB and BDNF in N2a cells under the indicated treatments. (**g**) Quantification showing that morphine increased total TrkB, p-TrkB and BDNF expression, whereas 4EGI-1 pretreatment attenuated these morphine-induced changes. *n* = 4, * *p* < 0.05, ** *p* < 0.01.

To examine the involvement of eIF4E-dependent translation in morphine-induced apoptotic injury, N2a cells were treated with 4EGI-1, an inhibitor of the eIF4E/eIF4G interaction, either alone or in combination with morphine, as illustrated in Figure 5a. DAPI staining showed that morphine treatment induced evident nuclear condensation and fragmentation, whereas combined treatment with 4EGI-1 further increased the number of apoptotic nuclei (Figure 5b). Consistently, cell viability analysis showed that 4EGI-1 alone had no significant effect (*F* = 1.849, *p* = 0.211), while morphine markedly reduced cell viability; this reduction was further exacerbated by cotreatment with 4EGI-1 (Figure 5c, *F* = 5.607, *p* = 0.045).

Western blot analysis further demonstrated that morphine increased Bax expression (*F* = 13.925, *p* = 0.005), decreased Bcl-2 expression (*F* = 7.221, *p* = 0.025), and increased cleaved caspase-3 expression (*F* = 19.542, *p* = 0.002), further supporting apoptosis-related responses. These changes were strengthened by 4EGI-1 cotreatment, as reflected by an additional increase in Bax (*F* = 7.992, *p* = 0.020) and cleaved caspase-3 expression (*F* = 16.733, *p* = 0.003), whereas Bcl-2 remained at a low level without a further significant decrease (*F* = 2.231, *p* = 0.169) (Figure 5d,e). Given that morphine enhanced BDNF/TrkB signaling in N2a cells, we next examined whether this pathway was affected by 4EGI-1. Morphine significantly increased the protein expression of TrkB (*F* = 9.974, *p* = 0.012) and BDNF (*F* = 5.284, *p* = 0.047). Importantly, morphine also increased p-TrkB levels (*F* = 10.270, *p* = 0.011), indicating functional activation of TrkB-related signaling under morphine exposure. In contrast, cotreatment with 4EGI-1 significantly attenuated the morphine-induced increases in TrkB (*F* = 17.418, *p* = 0.002), p-TrkB (*F* = 11.319, *p* = 0.008) and BDNF (*F* = 21.001, *p* = 0.0013) (Figure 5f,g).

In summary, these results indicate that pharmacological disruption of eIF4E/eIF4G interaction aggravates morphine-induced apoptotic injury and attenuates morphine-induced increases in BDNF and TrkB expression in N2a cells. These findings suggest that eIF4E/eIF4G-mediated translation initiation may be involved in an adaptive response to morphine-induced cellular stress and may be associated with changes in BDNF/TrkB signaling, rather than serving as a purely pro-apoptotic signal.

### 2.6. Injection of 4EGI-1 into the mPFC Inhibited Morphine-Induced CPP

**Figure 6:**
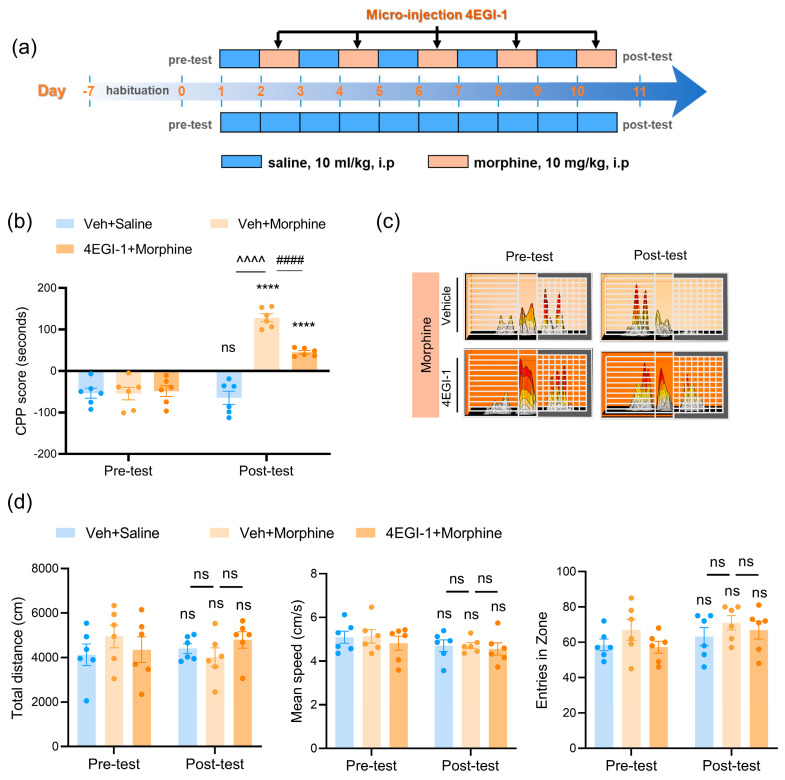
Injection of 4EGI-1 into the mPFC inhibited morphine-induced conditioned place preference without affecting motor activity. (**a**) Schematic illustration of the experimental design. Mice underwent habituation and pre-test, followed by conditioning and post-test. 4EGI-1 was microinjected into the mPFC before each morphine conditioning session during training. (**b**) Morphine significantly increased CPP score, whereas intra-mPFC administration of 4EGI-1 before morphine conditioning significantly attenuated this effect. *n* = 8, **** *p* < 0.0001 compared to the pre-test, ^^^^ *p* < 0.0001 compared to the Veh + Saline, #### *p* < 0.0001 compared to the Veh + Morphine. Veh, vehicle. (**c**) Representative heatmaps showing intra-mPFC administration of 4EGI-1 reduced occupancy in the morphine-paired chamber during the post-test. (**d**) Quantification of total distance traveled, mean speed and entries into each chamber during the pre-test and post-test phases, showing no significant differences among groups. *n* = 8, ns, not significant.

4EGI-1 was microinjected into the mPFC before each morphine conditioning session during the training phase of the CPP to examine whether eIF4E/eIF4G interaction in the mPFC is required for morphine-induced reward memory formation (Figure 6a). In the pre-test, no significant difference in CPP score was observed among the Veh + Saline, Veh + Morphine, and 4EGI-1 + Morphine groups, indicating comparable baseline chamber preference before conditioning. After conditioning, mice in the Veh + Morphine group exhibited a marked increase in CPP score compared with the Veh + Saline group (*F* = 64.737, *p* < 0.0001), confirming successful induction of morphine-associated place preference. Notably, intra-mPFC administration of 4EGI-1 significantly reduced the CPP score in morphine-treated mice relative to the Veh + Morphine group (*F* = 46.314, *p* < 0.0001), indicating that blockade of eIF4E/eIF4G interaction in the mPFC before morphine pairing impaired the formation of morphine-induced reward memory (Figure 6b). Representative heatmaps further supported this behavioral effect (Figure 6c).

To exclude the possibility that the reduced CPP score resulted from altered locomotor activity, locomotion-related parameters were further analyzed. No significant differences were detected among the three groups in total distance traveled or entries into each chamber during either the pre-test or post-test phases (Figure 6d). These findings indicate that the inhibitory effect of 4EGI-1 on CPP was not attributable to changes in general locomotor capacity or exploratory behavior.

### 2.7. Injection of 4EGI-1 into the mPFC of Mice Does Not Alter Locomotor Activity, Anxiety-like Indices, or Cognitive Performance

**Figure 7:**
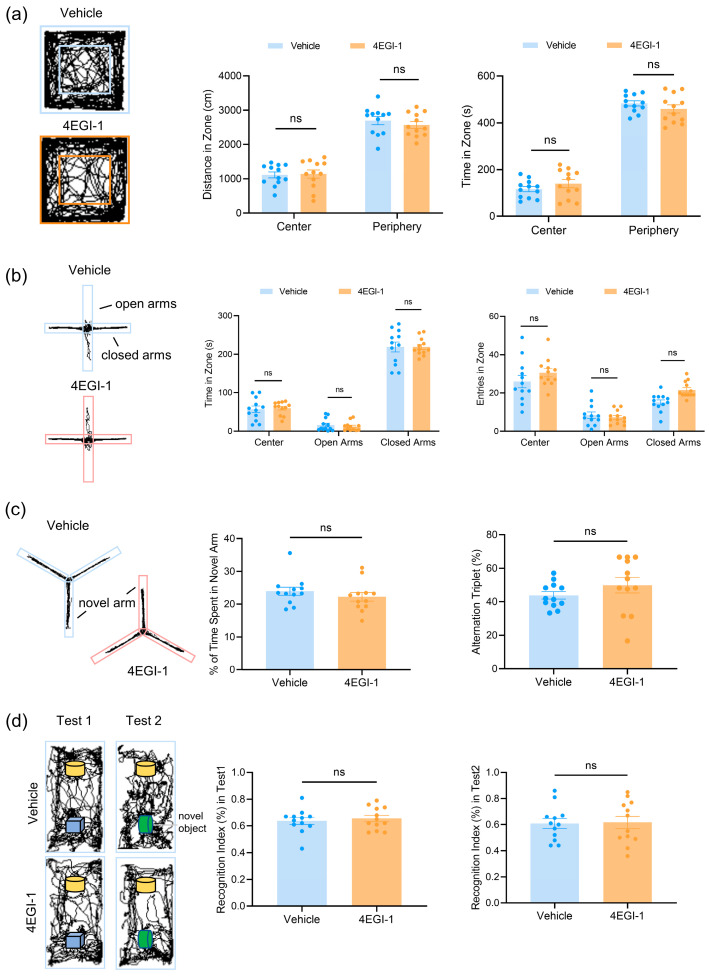
Injection of 4EGI-1 into the mPFC does not produce detectable changes in anxiety-like behavior or general cognitive performance in mice. (**a**) Left: Representative movement trajectories of OFT; right: quantitative analysis. Compared with the vehicle group, mice in the 4EGI-1 group showed no significant differences in either the distance traveled or the time spent in the center and peripheral zones. *n* = 12, ns, not significant. (**b**) Left: Representative movement trajectories of EPM; right: quantitative analysis. Compared with the vehicle group, the 4EGI-1 group showed no significant differences in either the time spent in each arm or the number of entries into each arm. *n* = 12, ns, not significant. (**c**) Left: Representative movement trajectories of YMT; right: quantitative analysis. No significant differences were observed between the 4EGI-1 and vehicle groups in either the time spent in the novel arm or the spontaneous alternation rate. *n* = 12, ns, not significant. (**d**) Representative exploration trajectories from the NOR test are presented on the left, while the quantitative analysis of the recognition index is shown on the right. No significant differences were observed between the 4EGI-1 and vehicle groups in either test 1 or test 2. *n* = 12, ns, not significant.

Mice receiving intra-mPFC administration of 4EGI-1 were further subjected to a battery of behavioral tests to assess whether its effects on morphine-induced CPP were associated with nonspecific alterations in anxiety-like behavior or cognition. In the open field test (OFT), anxiety-like behavior was evaluated based on center-zone exploration, including the time spent and distance traveled in the center zone. 4EGI-1 did not significantly alter the distance traveled (center: *t* = 0.1733, *p* > 0.99; periphery: *t* = 0.8789, *p* = 0.7779) or time spent (center: *t* = 1.085, *p* = 0.5789; periphery: *t* = 1.086, *p* = 1.086) in either the center or peripheral zones compared with vehicle-treated mice (Figure 7a), indicating 4EGI-1 had no detectable effect on general exploratory behavior or anxiety-like behavior. In the elevated plus maze (EPM) test, anxiety-like behavior was mainly assessed by open-arm exploration, including the time spent in the open arms and the number of open-arm entries. No significant differences were observed between the vehicle and 4EGI-1 groups in the time spent in the center, open arms, or closed arms (center: *t* = 0.293, *p* > 0.99; open arm: *t* = 0.397, *p* > 0.99; closed arm: *t* = 0.003, *p* > 0.99), nor in the number of entries (center: *t* = 1.682, *p* = 0.306; open arm: *t* = 0.428, *p* > 0.99; closed arm: *t* = 2.385, *p* = 0.069) into these zones (Figure 7b). In the Y-maze test (YMT), 4EGI-1 treatment did not affect the percentage of time spent in the novel arm (*t* = 0.936, *p* = 0.360) or the spontaneous alternation rate (*t* = 1.189, *p* = 0.247) (Figure 7c), suggesting intact spatial working memory was not been impaired. Likewise, in the novel object recognition (NOR) test, the recognition index did not differ significantly between vehicle and 4EGI-1 groups in short-term memory test (test 1, *t* = 0.515, *p* = 0.612) and long-term memory test (test 2, *t* = 0.123, *p* = 0.903) (Figure 7d), indicating that object recognition memory remained unchanged.

These results indicate that intra-mPFC administration of 4EGI-1 does not produce detectable changes in locomotor activity, anxiety-like indices assessed by OFT and EPM, or general cognitive performance assessed by YMT and NOR under the present experimental conditions. Therefore, the inhibitory effect of 4EGI-1 on morphine-induced CPP is unlikely to be explained by nonspecific impairments in the tested behavioral domains, supporting the behavioral specificity of this effect.

## 3. Discussion

### 3.1. Morphine-Induced Reward Memory Is Accompanied by Apoptotic Alterations in the mPFC

Drug addiction is fundamentally characterized by the formation and persistent maintenance of aberrant reward memory associated with drug-related environmental cues [30]. As a key brain region involved in contextual information integration, behavioral control, and reward memory regulation, the mPFC is critically positioned in addiction-related neuroplasticity [31]. Therefore, clarifying the molecular and cellular alterations that occur in the mPFC following morphine exposure is of particular importance for understanding the neurobiological basis of addiction-associated memory.

In the present study, a morphine-induced CPP model was successfully established, and the observed behavioral changes were shown not to result from alterations in general locomotor activity, but rather to reflect the stable formation of morphine-associated reward memory. TUNEL staining and Bax/Bcl-2 analysis further demonstrated that morphine exposure markedly enhanced apoptosis-related responses in the mPFC. Consistently, in vitro experiments showed that morphine reduced N2a cell viability in a dose-dependent manner, induced nuclear condensation and fragmentation, and was accompanied by increased Bax expression and decreased Bcl-2 expression. These findings indicate that morphine not only promotes reward memory formation but also triggers stress-related injury and apoptotic programs in neuron-like cells.

Previous studies have shown that opioid exposure can induce apoptosis-related alterations in the nervous system, although the available evidence has largely focused on general neurotoxicity or withdrawal-related injury. Arababadi MK et al. reported that opioid treatment induced apoptosis in Jurkat cells and, based on qPCR analysis, was associated with increased expression of the pro-apoptotic molecule Bax, along with increased expression of the anti-apoptotic and apoptosis-related molecules Bcl-2, Dffa, and Nol3 [32]. In addition, another study showed that morphine increased the Bax/Bcl-2 ratio and caspase-3 levels in the nucleus accumbens [33]. Collectively, these findings support the notion that morphine is capable of inducing molecular remodeling related to programmed cell death. However, for the mPFC, a brain region critically involved in reward memory and behavioral regulation, systematic evidence remains lacking as to whether apoptosis-like responses are directly engaged in the formation of morphine-associated reward memory.

The present study not only confirms that morphine induces CPP formation, but also shows that this process is accompanied by increased TUNEL-positive cells, elevated Bax expression, and reduced Bcl-2 expression in the mPFC. Combined with the findings from N2a cells, these results indicate good in vivo and in vitro consistency in morphine-induced apoptosis-like responses. More importantly, this apoptotic phenotype was further linked to increased eIF4E expression and altered BDNF/TrkB signaling. Notably, blockade of the eIF4E/eIF4G interaction attenuated morphine-induced CPP, but simultaneously aggravated cellular injury and weakened BDNF/TrkB upregulation. These findings suggest that apoptosis-related alterations in the mPFC are not merely a byproduct of opioid neurotoxicity, but may instead constitute a cellular stress context associated with the aberrant consolidation of reward memory. At the same time, eIF4E-dependent translation may play an adaptive protective role following morphine exposure.

### 3.2. eIF4E Is Responsive to Morphine Exposure and May Not Function as a Purely Pro-Apoptotic Factor

eIF4E is a key rate-limiting factor in cap-dependent translation initiation [34]. Upon binding to eIF4G, it forms the eIF4F complex and serves as a critical regulatory node controlling the translation efficiency of specific mRNAs [35]. Increasing evidence has shown that eIF4E/eIF4G-mediated translational initiation not only contributes to synaptic plasticity and learning and memory [36], but is also closely associated with neuronal stress responses, injury repair, and cell fate determination [37]. Dysregulation of eIF4E-related signaling has also been implicated in neuropsychiatric and neurodevelopmental disorders associated with altered neuronal translation. On this basis, the present study further examined the alteration and potential role of eIF4E following morphine exposure.

The results showed that morphine treatment increased neuronal eIF4E expression in both mPFC tissue and N2a cells. At first glance, the concurrent increase in eIF4E expression and apoptosis might suggest a pro-apoptotic role. However, functional intervention experiments revealed a more complex biological profile. Pharmacological disruption of the eIF4E/eIF4G interaction by 4EGI-1 further aggravated morphine-induced loss of cell viability, increased the number of apoptotic nuclei, and further elevated Bax expression. At the same time, intra-mPFC administration of 4EGI-1 in vivo markedly suppressed morphine-induced CPP formation. The novelty of the present study lies in connecting eIF4E/eIF4G-mediated translational regulation with both morphine-induced reward memory and apoptosis-related cellular responses, accompanied by changes in BDNF and TrkB expression.

Collectively, eIF4E upregulation more likely represents an adaptive protective response under morphine-induced stress. In drug-stimulated neurons, eIF4E may enhance the translation of a subset of mRNAs related to cell survival, repair, or plasticity, thereby helping maintain proteostatic balance and buffer toxic injury. Because this protective effect is insufficient to fully offset the damage caused by morphine itself, the overall apoptotic level still increases. Therefore, eIF4E is more likely to function as an adaptive regulatory molecule recruited after cellular injury, rather than simply acting as an upstream inducer that drives apoptosis.

### 3.3. eIF4E-Dependent Translation Links Reward Memory Formation, Adaptive Cellular Protection and BDNF/TrkB-Related Molecular Changes

4EGI-1 exhibited a seemingly paradoxical yet internally consistent dual effect in vivo and in vitro. Behavioral experiments showed that intra-mPFC administration of 4EGI-1 markedly suppressed morphine-induced CPP formation, indicating that eIF4E-mediated translational initiation in the mPFC is involved in morphine-associated reward memory formation. Importantly, since CPP performance may be influenced by locomotor activity, anxiety-like behavior, and general cognitive function, we performed OFT, EPM, YMT, and NOR assays to evaluate these potential confounding factors. Intra-mPFC 4EGI-1 did not detectably affect locomotion, anxiety-like behavior, spatial working memory or object recognition memory. Therefore, the attenuation of morphine-induced CPP by 4EGI-1 is unlikely to be explained by impaired motor ability, altered anxiety-related exploration, or broad cognitive dysfunction. Nevertheless, because CPP behavior can also be modulated by reward valuation, motivation, and cue-related emotional salience, these possibilities cannot be fully excluded and should be addressed in future studies. In contrast, in vitro experiments demonstrated that 4EGI-1 did not alleviate morphine-induced cellular injury, but instead further aggravated the apoptotic phenotype, as evidenced by a greater reduction in cell viability, an increased number of apoptotic nuclei, and a further elevation of Bax expression. These findings indicate that, in the context of morphine exposure, eIF4E-dependent translational control is not solely involved in aberrant memory formation, but is also closely associated with the adaptive maintenance of cellular homeostasis under stress.

This dual role is not necessarily contradictory. It can be explained by the broad yet transcriptionally selective role of eIF4E/eif4g in mediating translation initiation. The formation of reward memory depends on protein synthesis, particularly the rapid translation of synaptic plasticity-related proteins within specific temporal windows [38,39]. Our previous work showed that disruption of the eIF4E/eIF4G interaction suppresses the synthesis of multiple synaptic plasticity-related proteins, thereby attenuating morphine-associated reward memory [40]. At the same time, eIF4E-dependent translation may also contribute to the maintenance of survival-related molecules, thereby buffering morphine-induced cellular stress and injury. In the present study, morphine treatment significantly increased BDNF and TrkB expression in both the mPFC and N2a cells, whereas cotreatment with 4EGI-1 attenuated these increases in vitro. Importantly, the additional detection of p-TrkB in mPFC provides a functional pathway activation readout for interpreting these molecular changes. Given the established roles of BDNF/TrkB signaling in neuronal survival, synaptic plasticity, and addiction-related neural remodeling [23,41], these findings suggest a potential association between eIF4E-dependent translational control and the regulation of BDNF/TrkB-related molecules under morphine-induced cellular stress. Nevertheless, although TrkB phosphorylation provides stronger evidence than total protein expression alone, it does not fully establish causal necessity. The present study did not directly manipulate BDNF/TrkB signaling using TrkB inhibitors, BDNF neutralization, or genetic intervention. Therefore, the current data support functional activation and association of BDNF/TrkB-related signaling, but cannot prove that this pathway is required for eIF4E-dependent regulation of morphine-induced reward memory or apoptosis-related responses. Future studies using pathway-specific manipulation will be necessary to clarify the causal role of BDNF/TrkB signaling in this process.

In summary, the present findings indicate that eIF4E-dependent translational regulation has dual significance in the neurobiological response to morphine. On the one hand, it may contribute to morphine-associated reward memory formation; on the other hand, it may help maintain survival-related molecular signaling under conditions of cellular stress, thereby exerting an adaptive protective effect. These observations suggest that addiction-related molecular processes do not always act in a unidirectional manner to promote pathological progression, but are often accompanied by endogenous compensatory responses that help preserve cellular homeostasis. The current data support an association between eIF4E-dependent translational regulation, reward memory formation, and apoptosis-related cellular stress. Future studies assessing synaptic protein expression, dendritic spine remodeling, electrophysiological changes, and long-term potentiation will be required to determine whether eIF4E-dependent translation contributes to morphine-induced maladaptive plasticity.

Several limitations should also be acknowledged. First, the current evidence for the protective role of eIF4E-dependent translation is derived mainly from the N2a cell model and therefore may not fully recapitulate the responses of mature neurons in vivo. Second, although 4EGI-1 is widely used to disrupt the eIF4E/eIF4G interaction, it may also affect the broader cap-dependent translation initiation process. Therefore, the aggravated apoptosis observed after 4EGI-1 treatment cannot be attributed exclusively to an eIF4E-specific effect, but may also partly result from secondary cellular stress caused by general translation inhibition. Third, although the present data demonstrate clear associations among eIF4E/eIF4G-mediated translation initiation, apoptotic changes, and BDNF/TrkB alterations, due to the lack of manipulation of the BDNF/TrkB signaling function, it is still unclear whether the morphine-induced apoptotic response or CPP formation requires this pathway. Another limitation is that the present study analyzed the mPFC as an integrated region without distinguishing between the prelimbic and infralimbic subregions, which may have distinct roles in drug-associated memory and emotional regulation. Future studies using eIF4E-specific genetic manipulation, BDNF/TrkB pathway inhibition or activation, and rescue experiments are needed to better define the stage-specific role of eIF4E in morphine-induced neuronal injury and reward memory formation, which also requires further validation using subregion-targeted approaches.

## 4. Materials and Methods

### 4.1. Animals

Male C57BL/6J mice aged 8–10 weeks and weighing 20–25 g were supplied by the Experimental Animal Center of Xi’an Jiaotong University. All animals were maintained in a specific pathogen-free (SPF) environment under controlled temperature and humidity conditions with a 12 h light/dark cycle, with food and water available ad libitum. Before the initiation of the experiments, the mice were acclimated to the animal room for one week. To minimize stress during subsequent procedures, animals were gently handled daily, including grasping, stroking, and sham intraperitoneal injection during the acclimation period. Behavioral testing was consistently performed during the light cycle in order to minimize potential interference from circadian rhythms. Mice were randomly allocated to experimental groups. All procedures involving animals complied with institutional guidelines for the care and use of laboratory animals and received approval from the Biomedical Ethics Committee of Health Science Center of Xi’an Jiaotong University.

### 4.2. Drug and Reagents

For pharmacological treatment, morphine hydrochloride obtained from a commercial supplier (Shenyang First Pharmaceutical Factory, Shenyang, China) was formulated in sterile physiological saline before the experiment. Morphine was administered to mice by intraperitoneal injection at 10 mg/kg body weight, a dose widely used in rodent conditioned place preference paradigms to establish morphine-associated reward memory and addictive-like behavioral responses. Animals assigned to the control group received the same injection volume of saline alone. 4EGI-1 (HY-19831, 10 mM in DMSO, MedChemExpress, Monmouth Junction, NJ, USA) was prepared freshly before each experiment and diluted to a working concentration of 80 μM according to previous studies [40]. 4EGI-1 was administered intraperitoneally 30 min before morphine injection. Every dosing solution was newly made each experimental day and brought to room temperature before injection.

### 4.3. Behavioral Experiments

#### 4.3.1. Conditioned Place Preference

Morphine-associated rewarding memory was assessed in mice using the conditioned place preference paradigm. Mice were randomly distributed into a morphine-treated cohort or a saline-treated control cohort. The behavioral device contained three communicating compartments, including two larger side chambers used for conditioning and a smaller central chamber serving as the neutral zone. The two conditioning chambers differed in sensory features, including wall appearance and floor pattern, to allow context–drug association. The CPP procedure consisted of habituation, pre-test, conditioning, and post-test phases. On the pre-test day, mice were allowed to freely explore all compartments for 15 min, and baseline chamber preference was recorded. During the conditioning phase, mice received morphine or saline on alternating sessions for 10 consecutive days and were confined to the corresponding paired chamber after each injection. On the post-test day, mice were tested in a drug-free state and allowed to freely explore all compartments. Locomotor activity and chamber preference were monitored with Smart 3.0 software (Panlab SL, Barcelona, Spain), which automatically extracted parameters including total movement distance, residence time in each chamber, and transition frequency between compartments. The CPP score was calculated as the time spent in the morphine-paired chamber minus the time spent in the saline-paired chamber. Experimental procedures were established on the basis of previously reported protocols with slight adjustments for the present study [42].

#### 4.3.2. Open Field Test

The OFT was employed to assess spontaneous locomotion and anxiety-related behavior in mice. For testing, each animal was introduced into the middle of a square open-field chamber (45 cm × 45 cm × 45 cm) and permitted to move freely for 10 min under constant lighting conditions. Locomotor behavior was tracked and analyzed with Smart 3.0 software after the arena had been digitally segmented into center and border regions. The total distance traveled was used as an index of general locomotor activity. Anxiety-like behavior was evaluated mainly by the time spent in the center zone and the distance traveled in the center zone, with reduced center exploration interpreted as increased anxiety-like behavior. The time spent and distance traveled in the peripheral zone were also recorded as complementary parameters. The chamber was disinfected with 75% ethanol after each trial to eliminate residual olfactory cues.

#### 4.3.3. Elevated Plus Maze

Anxiety-like behavior was further examined using the EPM. The maze was composed of two opposing open arms (30 cm × 5 cm) and two opposing enclosed arms (30 cm × 5 cm × 15 cm), with the entire apparatus positioned 50 cm above the ground. At the start of the test, each mouse was introduced onto the central platform with its head oriented toward an open arm and was then permitted to explore the maze for 5 min. Animal movement was monitored and subsequently analyzed with Smart 3.0 software. The primary anxiety-related indices were the time spent in the open arms and the number of open-arm entries, with reduced open-arm exploration considered to reflect increased anxiety-like behavior. The time spent in the closed arms, the number of closed-arm entries, and the time spent in the central platform were also recorded as complementary behavioral parameters. Following each session, the maze was wiped with 75% ethanol to remove residual olfactory cues.

#### 4.3.4. Y-Maze Test

Spatial working memory was assessed using the Y-maze behavioral task. The maze consisted of three arms of identical dimensions (30 cm × 6 cm × 15 cm), arranged symmetrically at 120° relative to one another. In the acquisition session, access to one arm was blocked, and each mouse was permitted to explore the remaining two arms for 10 min. Following a 2 h retention interval, the mice were returned to the maze for a 5 min trial with all arms accessible. Animal movement was tracked and subsequently processed using Smart 3.0 software. A spontaneous alternation event was defined as sequential entry into all three arms without repetition. The alternation rate was calculated according to the following formula: spontaneous alternation (%) = [actual alternations/(total arm entries − 2)] × 100.

#### 4.3.5. Novel Object Recognition

The NOR test was performed to assess recognition memory in mice. Animals were habituated to an empty arena (25 cm × 25 cm × 30 cm) for 10 min per day for two consecutive days. During the training session, two identical objects were placed in the arena and the mice were allowed to explore for 5 min. After 3 h, one familiar object was replaced with a novel object for the short-term memory test, and exploration was recorded for 5 min. At 24 h after training, a different novel object was introduced to evaluate long-term memory. Recognition memory was evaluated using the recognition index: RI (%) = time spent exploring the novel object/(time spent exploring the novel object + time spent exploring the familiar object) × 100%. The arena and objects were cleaned with 75% ethanol between trials.

### 4.4. Sample Preparation and TUNEL Staining

Neuronal apoptosis in the mPFC was evaluated by terminal deoxynucleotidyl transferase dUTP nick-end labeling staining performed in accordance with the supplier’s protocol. Paraffin brain sections were first dewaxed and rehydrated, then subjected to Proteinase K digestion before incubation with the TUNEL equilibration buffer and labeling solution. Following rinsing in PBS, the sections were counterstained with DAPI, coverslipped using an anti-fade mounting reagent, and visualized under fluorescence microscopy. Apoptotic cells within the mPFC were recognized by Alexa Fluor 594-derived red fluorescence, whereas cell nuclei were identified by DAPI staining.

### 4.5. Neuronal Protein Extraction

Total protein from mPFC tissue was prepared with N-PER™ Neuronal Protein Extraction Reagent (Cat. No. 87792; Thermo Fisher Scientific, Waltham, MA, USA) following the supplier’s recommended procedure. In brief, freshly dissected samples were washed in pre-chilled PBS and then homogenized on ice in extraction buffer containing protease and phosphatase inhibitor cocktails. Protein extraction was performed using a tissue-to-buffer proportion of 1:10 (*w*/*v*), as recommended by the manufacturer. Following mechanical disruption under cold conditions, the lysates were allowed to stand briefly and then clarified by centrifugation at 10,000× *g* for 10 min at 4 °C. The resulting supernatant was recovered as the total neuronal protein fraction and subjected to downstream analyses.

### 4.6. Western Blotting

Protein abundance in each sample was quantified with a bicinchoninic acid (BCA) assay kit. For Western Blotting, equivalent quantities of protein were combined with 5× sample buffer, heat-denatured at 95 °C for 5 min, resolved by SDS–polyacrylamide gel electrophoresis, and subsequently electrotransferred to PVDF membranes. After transfer, the membranes were incubated in TBST containing 5% skim milk for 1 h at room temperature to block nonspecific binding, followed by overnight exposure at 4 °C to the indicated primary antibodies at supplier-recommended dilutions: anti-eIF4E (66655-1-Ig, 1:15,000, Proteintech, Wuhan, China), anti-Bax (T40051, 1:1000, Abmart, Shanghai, China), anti-Bcl-2 (WanleiBio, WL01556, 1:1000), anti-cleaved caspase-3 (WL01992, 1:1000, WanleiBio, Shenyang, China), anti-TrkB (WL00839, 1:1000, WanleiBio, Shenyang, China), anti-p-TrkB (WL02988, 1:500, WanleiBio, Shenyang, China), anti-BDNF (T55577, 1:1000, Abmart, Shanghai, China), and anti-GAPDH (SA00001-1, 1:5000, Proteintech, Wuhan, China). Following TBST rinses, the blots were incubated with the appropriate secondary antibodies (1:5000) for 2 h at ambient temperature. Immunoreactive signals were developed using an enhanced chemiluminescence detection system. Densitometric analysis was performed in ImageJ software (Version 1.54p), and target protein levels were normalized against GAPDH.

### 4.7. Cell Culture and Treatments

The murine neuroblastoma-derived Neuro-2a cell line (N2a; RRID: CVCL_0470) was obtained from Procell (Cat. No. CL-0168; Wuhan, China). Cells were maintained in DMEM containing 10% fetal bovine serum and 1% penicillin–streptomycin under standard culture conditions (37 °C, 5% CO_2_).

To establish an in vitro morphine-induced cellular injury model, N2a cells were incubated with morphine at final concentrations of 10, 100, or 1000 μM for 48 h. This concentration range was selected based on previous in vitro studies in neuronal or neuron-like cells. For pharmacological disruption of the eIF4E–eIF4G complex, cultures were exposed to 4EGI-1 (50 μM) for 6 h.

### 4.8. DAPI Staining Assay

N2a cells cultured on glass coverslips were fixed in 4% paraformaldehyde at ambient temperature, rinsed with PBS, and then stained with DAPI solution for 5–10 min under light-protected conditions. Following additional PBS washes, the coverslips were mounted with an anti-fade medium and examined by fluorescence microscopy. Cells undergoing apoptosis were recognized according to characteristic nuclear alterations, including condensed chromatin, pyknotic nuclei, and fragmented nuclear structures.

### 4.9. Cell Viability Assay

Cell survival was evaluated using a Cell Counting Kit-8 assay (CCK-8; WanleiBio, WLA074a, Shenyang, China). N2a cells were plated in 96-well culture plates and maintained for 12 h to permit attachment prior to morphine exposure. At the end of the treatment period, 10 μL of CCK-8 reagent was added to each well, followed by incubation at 37 °C for 1 h. Optical density at 450 nm was recorded using a Thermo Fisher microplate reader. The viability of each treatment group was expressed relative to the control condition. Three independent biological replicates were included for each group.

### 4.10. Quantitative Real-Time PCR

Total RNA was extracted using the RNAfast1000 kit (Pioneer Biotechnology, Shaanxi, China). RNA quantity and purity were measured with a NanoDrop 2000c spectrophotometer (Thermo Fisher Scientific, Waltham, MA, USA). cDNA was synthesized using Hifair III 1st Strand cDNA Synthesis SuperMix for qPCR (Yeasen Biotechnology, Shanghai, China). Quantitative PCR was performed with the SYBR Green Premix Pro Taq HS qPCR Kit (Accurate Biology, Hunan, China), and relative expression levels were determined by the 2^−ΔΔCt^ method using GAPDH as the internal reference. Primer sequences are provided in Table 1.

### 4.11. Intracranial Cannula Implantation and Microinjection

A customized intracranial cannula system (RWD Life Science, Shenzhen, China), including a guide cannula (c = 3.2 mm), an internal injection cannula (c = 3.7 mm), a locking cap, and connecting tubing (62320), was used for local drug delivery into the mPFC. Mice underwent stereotaxic surgery under anesthesia, and the guide cannula was bilaterally implanted 0.5 mm above the mPFC at the following coordinates: AP, +1.98 mm; ML, ±0.30 mm; DV, −1.70 mm. Stainless steel screws were inserted into the skull for stabilization, and the cannula assembly was secured with dental cement. After surgery, mice were allowed to recover on a heating pad, received penicillin intraperitoneally to prevent infection, and were monitored daily.

During microinjection, a 1.0 μL microsyringe was connected to the internal injection cannula via tubing prefilled with glycerol to eliminate air bubbles and ensure stable infusion. The 4EGI-1 was diluted to a working concentration of 80 μM and administered at a rate of 50 nl/min using a microinfusion pump. 200 nl 4EGI-1 was injected on each mPFC side. After the administration was completed, the internal cannula was left in place for 2 min to allow diffusion before withdrawal, and the guide cannula was sealed with a cap. The dose and timing were selected to target the conditioning phase rather than the expression phase of CPP.

After completion of behavioral testing, mice were deeply anesthetized, and their brains were collected for verification of cannula placement. Coronal brain sections containing the mPFC were prepared, and the injection sites were identified according to the cannula track and anatomical landmarks based on the mouse brain atlas.

### 4.12. Statistical Analysis

Statistical processing was carried out using IBM SPSS Statistics 27.0 and GraphPad Prism 9.0.0. All quantitative results are expressed as the mean ± standard error of the mean (SEM). Data normality was assessed using the Shapiro–Wilk test, and homogeneity of variance was examined using Levene’s test before statistical comparisons. All the data were normally distributed. Differences between the two groups were evaluated with an independent-samples *t*-test. For datasets involving multiple morphine concentrations, one-way ANOVA was employed, while two-way ANOVA was used for the remaining experiments. When multiple comparisons were required, Bonferroni’s post hoc correction was applied. Assumptions of data normality and variance homogeneity were examined prior to hypothesis testing. Statistical significance was defined as *p* < 0.05.

## 5. Conclusions

Morphine-induced reward memory formation is accompanied by apoptosis-related alterations in the mPFC, along with increased neuronal eIF4E expression and enhanced BDNF/TrkB expression. Pharmacological inhibition of the eIF4E/eIF4G interaction significantly affected morphine-induced CPP formation, apoptosis-related changes, and BDNF/TrkB expression. These findings suggest a potential functional association between eIF4E/eIF4G-mediated translational regulation, morphine-associated reward memory formation, cellular stress responses, and changes in BDNF and TrkB expression. This study provides new experimental evidence for understanding the molecular mechanisms of morphine addiction and identifying potential therapeutic targets.

## Figures and Tables

**Table 1:** Sequences of primers used for qPCR.

Gene	Primer Sequence
TrkB	F: GGTGGCTGTGAAGACGCTGAAG
	R: AATGTGCTCGTGCTGGAGGTTG
BDNF	F: CGACGACATCACTGGCTGACAC
	R: GAGGCTCCAAAGGCAC-TTGACTG
GAPDH	F: TCTCCTGCGACTTCAACA
	R: TGTAGCCGTATTCATTGTCA

## Data Availability

The original contributions presented in this study are included in the article. Further inquiries can be directed to the corresponding author.

## Abbreviations

The following abbreviations are used in this manuscript:

Bax	Bcl-2-associated X protein
Bcl-2	B-cell lymphoma/leukemia-2
BDNF	brain-derived neurotrophic factor
CPP	conditioned place preference
eIF4E	eukaryotic translation initiation factor 4E
eIF4G	eukaryotic translation initiation factor 4G
EPM	elevated plus maze
mPFC	medial prefrontal cortex
NAc	nucleus accumbens
NOR	novel object recognition
OFT	open field test
TrkB	tropomyosin receptor kinase B
YMT	y-maze test
